# Encounters in the Swedish health and welfare sector: experiences of people identifying as LGBTQ+

**DOI:** 10.1186/s12889-025-25795-2

**Published:** 2025-12-01

**Authors:** Heléne Appelgren Engström, Anna-Lena Almqvist

**Affiliations:** 1 School of Health, Care and Social Welfare, Mälardalen University, Västerås, Sweden; 2 Faculty of Arts and Social Sciences, Karlstad University, Karlstad, Sweden

**Keywords:** Caring encounters, Experiences, Healthcare, LGBTQ+, Uncaring encounters, Welfare sector

## Abstract

**Background:**

People identifying as LGBTQ + experience challenges when encountering the health and welfare sector. They experience heteronormativity and discrimination even though the health and welfare sector should value everyone equally and counteract discrimination. Research about the health and wellbeing of people identifying as LGBTQ + is mostly conducted in North America, and less research is conducted in Sweden; therefore, this study seeks to explore encounters with professionals in the health and welfare sector experienced by people identifying as LGBTQ+.

**Methods:**

This study used a qualitative method involving 22 persons identifying as LGBTQ+. Individual semi-structured interviews were conducted, and data was analysed with content analysis.

**Results:**

The main category, *Varying encounters in the health and welfare sector*, includes the two generic categories, *Caring encounters* and *Uncaring encounters*. Caring encounters include three sub-categories: *Welcoming environment*,* Respectfulness and Competent professionals*. Uncaring encounters includes three sub-categories: *Heteronormative assumptions*,* Intimidating behaviours*, and *Experiences of discrimination*.

**Conclusions:**

Professionals in the health and welfare sector need to demonstrate that they support inclusion by having LGBTQ + competence and knowledge. More effort is needed to encourage professionals in the health and welfare sector to understand individuals identifying as LGBTQ+. Professionals need to be given the prerequisites to develop guidelines for caring encounters and competence in using inclusive language.

**Supplementary Information:**

The online version contains supplementary material available at 10.1186/s12889-025-25795-2.

## Background

The UN’s Sustainable Development Goals (SDG) highlight the importance of ensuring healthy lives and promoting both well-being and mental health (SDG 3). Furthermore, SDG 10 states, among other things, that the aim is to reduce inequality within and between countries. SDG 10 calls for action to reduce inequalities in health care and promote social inclusion and non-discrimination. Regardless of these SDG goals, people identifying as lesbian, gay, bisexual, transgender, queer and other gender identities (LGBTQ+) still face challenges and barriers when accessing and receiving equitable health care and welfare support. As noted by Harvey: “We all have an obligation to push public health towards more inclusive language for LGBTQ + individuals if we want to make any progress in advancing health equity and producing high-quality science” (1 p. 17).

Sexuality affects most people throughout their lives and may have a major impact on general health, quality of life, self-esteem, and personal relationships. A systematic review [1] reports that persons identifying as LGBTQ + still experience discrimination and prejudice in both health care and social care despite the availability of appropriate training programmes for staff, recommendations, best practice examples, and statements of good intent [1]. Another systematic review describes sexual minority women’s health experiences in the UK, and the findings highlighted significant barriers, including heteronormative assumptions, perceptions and experiences of negative responses to coming out, ignorance and prejudice from healthcare professionals, and barriers to raising concerns or complaints. Moreover, little information was available about bisexual and trans women’s issues [2].

Furthermore, another review states that health care professionals in the UK lack knowledge about LGBTQ + identities and terminology, especially concerning transgender and non-binary individuals [3]. A recently published scoping review shows a lack of critical evidence about the mental and physical health needs, safety, and well-being of sexual and gender minority young people in low and lower-middle-income countries [4]. On the other hand, one study shows that evidence-based education focusing on communication and knowledge about LGBTQ + issues contributed to nurses reported improved ability to use appropriate language and higher confidence when providing care to patients identifying as LGBTQ+ [5].

People identifying as LGBTQ + experience significant physical and psychosocial health issues and concerns, and encounter barriers when accessing healthcare services. Literature reviews show that people identifying as LGBTQ + encounter heteronormativity in healthcare [6–8] and even stigma, as well as limited access to health services and inadequate knowledge among health care providers [6].

Youths identifying as LGBTQ + are largely overrepresented in the child welfare system. Research also shows that LGBTQ children are overrepresented in the foster care system in the United States. These children are also at higher risk for homelessness and suicide [9]. The experiences of LGBTQ youths have largely been overlooked. They often encounter a number of challenges and disparities as they navigate the child welfare system. Many report experiences of discrimination, marginalisation, and an overall lack of acceptance [10]. A Swedish study investigated digital marketing for state care providers and found that LGBTQ + issues are largely invisible. Only 20 of approximately 1,000 state care providers worked with or had competence in LGBTQ + issues [11].

A qualitative study explored LGBTQ + patients’ experiences of their encounters with physicians, and found that physicians making assumptions about gender, using incorrect pronouns, and not recognising heterogeneity within the transgender community were all issues negatively affecting the encounters. Also, there were challenges and fears related to the willingness of patients to disclose their sexual orientation and/or gender identity, and finally, there were experiences of discrimination [12].

Sweden has been a global leader in pioneering sexual and family policies and is ranked as one of the top ten countries in Europe regarding LGBTQ + rights [13]. The right to good health on equal terms is emphasised in Sweden [14, 15]. The Discrimination Act [15] states that all people have equal value and rights, and it is forbidden to discriminate based on ethnicity, religion or belief, disability, gender, gender identity or gender expression, sexual orientation or age.

Despite increased welfare, significant disparities in public health in Sweden have been reported. A majority of people identifying as LGBTQ + have good health but rate their health lower than the rest of the population. For example, lesbian women have poorer health and greater anxiety and worry than the rest of the population. Homosexuals and bisexuals report being subjected to abusive treatment [16].

In Sweden, bisexual people report a higher degree of ill-health compared to both homosexual and heterosexual people. Moreover, they rate their physical health as being worse than heterosexual peoples, but better than homosexual people’s. Bisexual women are at particular risk of mental illness, such as depression and anxiety, as well as self-harm, compared to homosexual and heterosexual women [17]. A Swedish survey shows that the general health of people identifying as trans is influenced not only by common health determinants but also by vulnerabilities unique to trans individuals. Most participants (*n* = 796) reported negative health care experiences, including trans-incompetence among health care professionals and postponing seeking health care due to previous experiences of transphobia [18].

To summarise, international and national reviews show that people identifying as LGBTQ + encounter challenges when they navigate the health and welfare system. A large part of the research has been carried out in North America [19]. Since Sweden is considered a relatively open and tolerant country, it is difficult to draw conclusions from that research. Therefore, research on the health and wellbeing of people identifying as LGBTQ + in Sweden needs to be strengthened. This study seeks to explore the experiences of people identifying as LGBTQ + in regard to encounters with professionals in the Swedish health and welfare sector. The results of this study are intended to contribute to concrete measures to promote inclusive encounters within the health and welfare sector.

## Methods

### Aim

The study aimed to explore encounters with professionals in the health and welfare sector experienced by people identifying as LBGTQ+.

### Design

This study is linked to an international project, “Marriage Attitudes in the 21 st Century” with different sub studies, and with the aim of gaining a more thorough understanding of the health and well-being of persons identifying as LGBTQ+, with a focus on relationships [20]. This is the first study based on Swedish data, with interview questions connected to the international project. This qualitative interview study reports on encounters with professionals in the health and welfare sector experienced by persons identifying as LGBTQ+. This article follows the consolidated criteria for reporting qualitative research [21].

### Setting and characteristics of participants

This study is based on individual semi-structured interviews conducted in Sweden during 2024. The inclusion criteria were adults, above the age of 18 years, who identified as LGBTQ+. The initial contact was made through various communities and organisations directly, as well as through social media. We used strategic selection, by reaching out to communities, organizations and social media for people who identify as LGBTQ+. These organizations and social media then informed about our study through their various channels. Those interested in participating then contacted the authors for further information, and if interested in participating, they made an appointment for an interview.

Twenty-two persons identifying as LGBTQ + participated in the study. They were between 21 and 71 years old. Eighteen participants had been born in Sweden, one in another European country and three in a country outside Europe. Six participants lived in a large city (Stockholm, Gothenburg or Malmö, the three biggest cities in Sweden), eight lived in a medium-sized city, and eight lived in a smaller city (< 10 000 inhabitants). Twelve participants had a university degree, two had higher vocational education, and eight had a high school diploma. Nine participants were working, one was working part-time while also on parental leave, and six were studying. Six participants were not in the labour force; they were, for example, retired, on sick leave or unemployed. Regarding the participants’ family situation, six were married, seven were cohabiting, five were in a relationship but lived separately, and four were single. Seven participants had children, and 15 did not have children.

### The interviews

Qualitative data may reveal complexity and provide a deeper understanding of a phenomenon. Semi-structured interviews have a low degree of standardisation and allow openness in the interview situation, making it easier to follow the interviewees’ storytelling [22]. Qualitative interviews also provide more opportunities for discovery than interviews with a high degree of control [23].

A semi-structured interview guide used for this study was developed within the framework of the previously mentioned international project. The participants were asked to talk about their experiences of encounters with professionals in the health and welfare sector. They were asked if they could give examples of good as well as suboptimal encounters, and if they had experienced discrimination within the health and welfare sector.

All but two interviews were conducted online. One of these two interviews took place in the participant’s home and the other at the participant’s workplace. The interviews lasted around one hour and were audio-recorded and then transcribed verbatim. Both authors conducted the interviews and both authors have experiences in conducting qualitative studies and interviews.

### Data analysis

We deemed a qualitative content analysis suitable to analyse the data. Content analysis according to Elo and Kyngäs [24] comprises three steps: preparation, organisation, and reporting. In the first step, preparation, we read the transcribed interviews several times to become familiar with the data. In the next step, organisation, we identified codes describing encounters in the health and welfare sector. Then we compared the codes in an ongoing discussion between us authors. We first grouped these codes into the generic categories of *Caring encounters* and *Uncaring encounters* and then grouped into sub-categories describing similarities and differences in encounters in the health and welfare sector. See Table 1 for an example of the analysis process. We both read and commented on the analysis and the final findings. In the last step, reporting, we reported the findings through the main category, *Varying encounters in the health and welfare sector*, with the two generic categories, *Caring encounters* and *Uncaring encounters*, with six sub-categories describing participants’ experiences, with clarifying quotes in each sub-category. The participants’ names are not revealed to ensure confidentiality.

**Table 1:** An example of the analysis process

Codes	Sub-category	Generic category
Rainbow flags	Welcoming environment	
Gender-neutral toilets		
Being friendly	Respectfulness	Caring encounters
Treated like anyone else		
Knowledge	Competent professionals	
Inclusive language		

### Ethical considerations

An ethical application for the interview study on which this paper is based was approved by the Swedish Ethical Review Authority with the reference number 2023–06481-01. The study has been guided by ethical principles and recommendations from the Swedish Research Council [25], which emphasise that participation in research is voluntary and should always be based on informed consent. The participants received written and verbal information about the study and had the opportunity to ask questions about it. Furthermore, they were informed about their rights not to answer all questions and to withdraw their participation at any time. All the participants agreed to participate, identified as LGBTQ + and were above the age of 18, thus adults.

## Findings

In this study, as regards people identifying as LGBTQ+, their experiences of encounters with professionals in the health and welfare sector can be described as *Varying encounters in the health and welfare sector.* These varied encounters are experienced by some participants, on the one hand, as welcoming and respectful encounters with competent professionals. On the other hand, some participants also experienced encounters where they were met with heteronormative assumptions, intimidating behaviours and experiences of discrimination. The findings describe a range of varied experiences from the health and welfare sector. All participants reported various experiences from the healthcare sector, while some participants had no experience from the welfare sector. The health sector includes experiences of encounters in healthcare and dentistry. The welfare sector includes experiences of encounters with employment services, social services, education and the police.

The findings are presented through the main category, *Varying encounters in the health and welfare sector*, with two generic categories: *Caring encounters* and *Uncaring encounters* (see Table 2).

**Table 2:** Overview of the results

Sub-category	Generic category	Main category
Welcoming environment		
Respectfulness	Caring encounters	Varying encounters in the
Competent professionals		health and welfare sector
Heteronormative assumptions		
Intimidating behaviour	Uncaring encounters	
Experiences of discrimination		

### Caring encounters

The category, Caring encounters, pertains to participants’ experiences of good encounters in the health and welfare sector. These caring encounters are described in the three sub-categories: *Welcoming environment*,* Respectfulness*, and *Competent professionals.*

#### Welcoming environment

A welcoming environment contributed to caring encounters. The participants’ experiences of a welcoming environment refer to laws and norms in society as well as the physical environment within the health and welfare sector.

Different laws and norms in society were considered to contribute to a safe environment in the health and welfare sector. The participants experienced that the discrimination law, have had a significant impact on norms in society. One participant had read about Swedish LGBTQ + rights and said, “*I moved to Sweden because I felt that I could live openly with my girlfriend here*” (i19). She experienced her encounter with the health and welfare sector as good and welcoming.

Regarding the physical environment, little things had great significance for whether the environment were perceived as welcoming or not. The rainbow flag was an important element in the environment and perceived as welcoming, according to the participants. One participant said, “*The rainbow flag signal a desire to be inclusive*” (i14). Posters that appealed to individuals who identify as LGBTQ + also contributed to an including and welcoming environment. The participants mentioned, for example, phone numbers for various organisations, which indicated the visibility of LGBTQ + individuals and their life situations. Furthermore, the participants highlighted the importance of information showing a positive attitude towards LGBTQ + issues. As one participant stated, “*What I think is positive is that when I have been to the gynaecologist*,* I have seen a lot. I have seen many*,* well*,* what do you call them? Well*,* posters about LGBTQ+*,* yes*,* health*,* and that it should be taken seriously and that it [the clinic] should be a safe place regardless of what gender you have. And you know*,* things like that*,* I think that is very positive*” (i11).

Other things that contributed to a physically welcoming environment included, for example, the presence of gender-neutral toilets. Encounters with professionals in the welfare sector were less frequently described by the participants in this study, but one said, “*I have had very few community interventions*,* have not had many contacts there*,* but it has been better than within the health care sector*,* I would say*” (i15). The participants experienced that they were not questioned in the welfare sector in the same way as in encounters with professionals in the health care sector.

#### Respectfulness

Respectfulness was important and contributed to a caring encounter. The participants’ experiences of respectfulness related to professionals being understanding and friendly and being treated like anyone else. Regarding professionals, being friendly and understanding, a respectful encounter also included professionals acting professionally. As one woman said, “*A good encounter means that you are treated with respect and goodwill. That no one raises their eyebrows or reacts to the fact that my wife is a woman*,* for example. It should just be obvious. The focus should be on my problem. That they behave professionally and respectfully*” (i17).

Another participant reflected on good encounters but wondered if this could be because no one saw that he identified as LGBTQ+. The participant said: “*I don’t feel that I have received any offensive treatment or anything like that*,* but I look like a completely ordinary person*,* so I think that in many ways*,* that kind of makes others treat me like anyone*,* like anyone in a positive way”* (i12).

The participants experienced that most encounters with professionals in both the health and welfare sectors were good. One participant, for example, emphasised that the Swedish Social Insurance Agency provides excellent encounters, while other participants expressed that both nurses and doctors they had met had been really nice people.

Respectfulness was described by the participants as professionals being friendly, and the participants being treated like anyone else, like a fellow human being. As one participant said, small things have great significance: “*Yes*,* I think it’s important to keep it somewhat simple*,* to show respect and all that. That’s enough. You don’t have to put on a show… Because I believe it can easily become a point of contention if you make a big deal out of it. If you treat people with respect*,* you will get very far”* (i18).

Furthermore, it was regarded as very positive when professionals only asked about partners, without mentioning gender. One participant said: “*I really appreciate that they don’t make assumptions—they see me as a woman*,* clearly*,* but they don’t assume I’m biologically female or that I have a male partner. Instead of asking if I have a boyfriend*,* they ask if I have a partner—or several partners—without mentioning gender. I think that’s great*,* because it’s not heteronormative. It shows they understand that I could have any kind of partner*,* of any gender*” (i11).

This participant clearly expresses the appreciation of professionals not making assumptions about the possible number of partners or gender. Another participant voiced satisfaction with the reception from her partner’s physician. She both verbally acknowledged her health issues as well as treated her without normative prejudices. The participant recounted:

*“Even though it was an uncomfortable issue*,* the doctor was calm and used humour*,* which helped us feel at ease. She didn’t question us when we said we both had the infection—no comments like*,* ‘Oh*,* but how could you both have it?’ or ‘You must have given it to each other.’ She just trusted what we said. I was especially grateful because I had a 70-year-old male doctor at the time and found it almost impossible to talk to him about anything gynaecological. This was a completely different experience”* (i6).

This statement exemplifies a reception that is not judgemental, and the participant makes a comparison with her older male doctor with whom health issues like these were not possible to discuss.

#### Competent professionals

Competent professionals contributed to caring encounters. Professionals in the health and welfare sector demonstrate competence by possessing the knowledge and skills to meet individuals who identify as LGBTQ + and by using inclusive language.

Professionals in the health and welfare sector with relevant knowledge and competence (about LGBTQ + issues) contribute to caring encounters. One participant talked about a positive healthcare encounter: *“I was having a Pap smear and was asked if I might be pregnant*,* etc. And usually they ask: Have you had sex in the last week? But then this midwife asked: Have you had sex that could lead to pregnancy in the last week? I thought that it was very inclusive and lovely and easy to answer”* (i1). Experiences of good encounters within trans care was understood as nothing strange because the staff operate on trans people and it is their special area, and thus they become very good at treatment, they know what to do, and they know the language.

Relevant knowledge and competence were experienced by the participants as: “*To be met with inclusive language and understanding and knowledge of the situation of homosexuals and thus be able to provide relevant information*” (i14). Professionals who provided relevant information and could answer their unique questions were experienced as competent.

Regarding inclusive language, this involves being met with open-ended questions, instead of making assumptions, and professionals using the correct pronouns. Several participants emphasised the importance of inclusive language and the use of the right pronouns to experience a good encounter. As one participant said: “*I have experienced good encounters in healthcare; they always use the right pronoun. The people I have met in healthcare have been good*” (i21). For other participating individuals, it mattered less whether they were met with the correct pronoun.

### Uncaring encounters

The category, uncaring encounters, pertains to participants’ experiences of suboptimal encounters in the health and welfare sector. These uncaring encounters are described through the three sub-categories: *Heteronormative assumptions*, *Intimidating behaviour*, and *Experiences of discrimination*.

#### Heteronormative assumptions

The participants experienced encountering heteronormative assumptions by professionals in both the health and welfare sectors. Heteronormative assumptions include professionals assuming everybody is heterosexual, participants being misgendered, and rude comments.

The participants in this study were usually misgendered by professionals they met in the health and welfare sector. As one participant said, “*No matter who you talk to*,* you always get the wrong gender. Always. I’m always called ‘she’. Also*,* it just feels like you’re making people uncomfortable. That’s probably why I think it is so hard to seek care”* (i13).

Being constantly faced with heteronormative assumptions was stressful and even led to participants not always seeking care. Professionals in the health and welfare sector lacked relevant knowledge about norms and gender, for example professionals were left speechless when they told them about their gender identity. The participants also felt that healthcare professionals even felt uncomfortable when they heard about their transsexual identity.

One participant talked about an encounter with a practitioner who asked if there was a chance that she could become pregnant and when she said no, the practitioner questioned how she could be so sure of that. The participant said that she had to explicitly state; “*it can’t be like that because I’m a lesbian”* (i12). The participants highlighted that professionals assume that the people they meet are living in a heterosexual relationship, which contributes to the person’s need to “come out” as homosexual in every new meeting. One participant said: “*They say that they believe that I am heterosexual*,* for example….I don’t know if that might be a blunt/rude comment*,* but it does create a bit of a strange situation if you correct someone and you see that person becoming completely bewildered or something like that*,* and they’re like*,* ‘Oh*,* really? Oh*,* what does that mean? Or*,* oh*,* how does that work?”* (i12). The participant said that professionals did not have experience of encountering trans persons.

#### Intimidating behaviours

Intimidating behaviours from professionals in the health and welfare sector contributed to uncaring encounters. Intimidating behaviours pertain to descriptions of participants’ experiences of professionals in the health and welfare sector that did not focus on participants’ problems but instead focused on what interested the professionals. Several participants experienced that professionals focused their questions on other issues than those the participants came to seek advice for.

One participant spoke about intimidating, irrelevant questions which were posed, that went far beyond the limits of professional behaviour: “*During my gender assessment*,* I met with a psychiatrist who asked if I was in a relationship*,* and I said I was with another guy. He thought it was very exciting that two guys were together and had a lot of questions about how that worked and so on. For example*,* he asked who was the active and who was the passive person in our relationship. It’s crazy that anyone would ask that*,* but the fact that it was a doctor working specifically in a gender dysphoria team… It’s just a complete lack of ethics*,* it’s screamingly obvious*” (i1).

This abuse of power might not be deliberate from the physician’s point of view, but the physician has the power to decide about gender-affirming surgery, which makes it even more difficult to set limits in the conversation and makes such comments even more intimidating.

The participant continued about the encounter: “*It became a difficult situation because he was also the one assessing me. In a way*,* he had control over my entire future. I responded very factually and briefly*,* but still gently: ‘We switch. Neither of us is the active one—we’re quite equal.’ Yet he kept going. ‘Oh wow*,* how exciting! What a great way you seem to have structured your relationship. Maybe everyone should be a bit more like that*’” (i1).

This quotation exemplifies a lack of respect for privacy by interrogating the subject about an issue that is irrelevant to the purpose of the visit. There is a power asymmetry since the person asking the question is also the physician who is in charge of deciding about surgery.

Intimidating behaviour includes when professionals ask irrelevant questions or express prejudices. One participant received a letter stating that he could have been exposed to gonorrhoea. At the doctor’s visit, the doctor talked about how gay men have so many sexually transmitted diseases, are careless, and bad at protecting themselves. The participant reported: “*The doctor said that homosexuals have more sexually transmitted diseases than other groups*,* but that was not true at the time because then there had been a super increase among heterosexuals linked to both the fact that there are more people in Sweden in heterosexual contexts who have serious diseases linked to being infected through sex*,* as well as chlamydia and gonorrhoea* (i14). The participant was very upset when talking about this situation.

Furthermore, some participants were sent to different doctors and wards to get the right treatment and care. They were met with questions about irrelevant issues relating to their bodies when seeking health care. One participant reported: “*One person whom I met questioned my appearance and thought that I had weird eyes and that I could not keep my eyes steady. I said that I could do that*,* but I was maybe stressed or nervous at the time… But yes*,* I don’t know if it is because I’m a trans person or not. It’s so difficult to know when you get that reception. Is it because a person can see that I’m LBTQ or…? I don’t know*” (i10).

This statement exemplifies a clear occurrence of an irrelevant statement not related to the health issue for which the participant was seeking care.

#### Experiences of discrimination

Experiences of discrimination contribute to uncaring encounters in the health and welfare sector. Some participants in this study had experiences of discrimination in these sectors. These experiences were undergone on both an organisational and a personal or professional level. The experiences of discrimination on an organisational level were, for example, that there were such long waiting times for gender-affirming care and that the care guarantee, which means a right to receive care within three months, did not apply to the type of care they needed. One participant said: *Discrimination*,* the issue is probably more that the care I need is so de-prioritised that it almost doesn’t exist*,* and when you do get in*,* there is an extremely long queue for care* (i20). The waiting time and queues for gender-affirming care were perceived as extremely long, and the participants felt like they were living in a vacuum while waiting for care.

Other participants had experiences of discrimination from health care professionals, i.e. discrimination on a personal or professional level. For example, one participant was not allowed to donate blood because of his sexual orientation. Another person with gender dysphoria who sought care was referred to a psychiatrist. Yet another participant spoke about an unprofessional experience with a dentist making assumptions about their health condition. The participant reported: *“I remember once going to the dentist after having been out all night*,* so my eyes were bloodshot. I didn’t have much money for food at the time*,* so I was rather thin and had red eyes. The dentist seemed to panic and referred me to Roslagstull [a hospital in Stockholm at the time with a focus on HIV patients]*, *though I didn’t really understand what was going on. But when I arrived at Roslagstull*,* it all became clear—there*,* both the dentist and the dental nurse were dressed in what looked like space suits. The dentist I had initially seen had apparently suspected that I had AIDS. He was terrified and refused to examine my mouth without gloves. When I arrived at the clinic*,* I was frightened and thought*,* “Where have I ended up?” But the staff there were very kind. They explained*,* ‘You’ve come to a clinic for patients [with HIV/AIDS]. That’s why we’re dressed like this. We understand that you’re not infected*,* but your dentist got scared…’ We’ll make sure to report that this is not an acceptable way to treat a patient. But now*,* let’s take a look at your teeth.’ So they examined me and then sent me home”* (i4).

In some clinical settings, trans individuals had been subjected to misdiagnosis and inappropriate referrals due to a lack of knowledge about gender identity among healthcare providers. In one case, a trans patient described how their disclosure of being transgender appeared to influence the clinical encounter, leading to an incorrect assumption of a psychiatric disorder.

This statement could also be related to age, with older participants having experience of HIV/AIDS, which is not the case for the younger ones, who merely have heard about it. Healthcare professional, upon learning they were a trans person, referred them to the outpatient psychiatry department with the suspicion of bipolar disorder. This occurred despite the participant explicitly explaining that they were experiencing gender dysphoria. The referral was subsequently rejected by the psychiatry department, who responded that “this person doesn’t need to see us.” The participant reflected on the experience, noting that “she [the doctor] didn’t know the difference” between gender dysphoria and bipolar disorder.

The situation highlights a broader concern about how trans identities are interpreted in medical records and consultations. The participant further articulated a fear that is not uncommon among trans patients: *“Will they read my record and see that I’m trans? Will that affect things?”* (i1). This apprehension reflects a perceived risk that one’s gender identity may unduly influence the care received, even when unrelated to the presented health issue.

The participant expressed the view that there had been assumptions about a need for psychiatric care, without listening to the patient. As a consequence, there was a fear that being transgender might affect other aspects of the care.

The participants reported having negative or positive experiences from encounters with professionals primarily in the health sector. The welfare sector was less represented. One participant recalled a not-so-positive experience with the police: “*It was a friend who abused me—physically and mentally. She moved into my home without permission and stalked me. When I reported it*,* I used gender-neutral words*,* and the police took me seriously—until I accidentally said ‘she.’ Their attitude shifted immediately. I wasn’t even finished with my story before they dismissed me. Later*,* when she physically attacked me in the street*,* the police said*,* ‘Well… it was a woman.’.* As the participant concluded: *“Had it been a man who attacked me*,* they would have investigated. But because it was a woman*,* it wasn’t worth their time*” (i8). The participant reported that once a gendered pronoun was mistakenly used, a shift in attitude was observed. This quote exemplifies how institutional responses to interpersonal violence can be heavily influenced by gendered assumptions and implicit bias.

## Discussion

This study aimed to explore encounters with professionals in the health and welfare sector experienced by people identifying as LGBTQ+. The participants reported experiences of varying encounters in the health and welfare sector. There was a large variation in the quality of encounters within both the health and welfare sector. These encounters are described using the categories of Caring and Uncaring encounters and will be discussed in relation to previous research and theoretical descriptions of encounters.

Caring encounters included a *Welcoming environment*,* Respectfulness*,* and Competent professionals.* The participants mentioned various laws and norms in society that contributed to a safe and welcoming environment within the health and welfare sector. As Sweden ranks as one of the top ten countries in Europe regarding LGBTQ + rights [13], it is encouraging that the participants felt that the laws made a difference. Overall, caring encounters are supported by the UN’s SDG 3, relating to good health and well-being. It is likely that SDG 10, which is aimed at reducing inequalities in health care and promoting social inclusion and non-discrimination, has also had an impact on norms and values in society. Furthermore, the participants in this study highlighted that both rainbow flags and gender-neutral toilets also contributed to a welcoming environment. Previous research states that health care professionals strive to create an inclusive environment [26]. The participants also appreciated professionals not making assumptions about gender or being judgmental. Research also highlights that educational efforts regarding suitable language and knowledge about LGBTQ + issues are important for boosting nurses’ confidence [5].

Most participants in this study described experiences of caring encounters with kind and respectful professionals in both the health and welfare sectors. From a theoretical perspective, caring encounters are dependent on competent and respectful professionals who genuinely care about the person they meet [27]. In Sweden, there is an emphasis on the right to good health on equal terms [14, 15]. The well-being of people identifying as LGBTQ + depends on professionals having relevant knowledge of LGBTQ + issues [5]. At the same time, the participants in this study expressed quite low expectations from professionals, as they reflected that a good encounter could be a result of professionals failing to notice that they identified as LGBTQ+. Previous research found that LGBTQ + individuals navigate complex health care experiences influenced by the salience of their identities, which can lead to both positive and negative interactions with healthcare providers [28].

Furthermore, the participants expressed the view that professionals in the health and welfare sector had knowledge and skills to meet people identifying as LGBTQ+, and the participants appreciated when professionals asked open-ended questions. Caring encounters are about having professional wisdom, which is a combination of knowledge and experience [27]. A good encounter and equitable care on equal terms require that professionals in the health and welfare sector know about the life situation and specific conditions of LGBTQ + individuals [29]. The Swedish National Board of Health and Welfare [30] states that professionals need to have LGBTQ + competence. Otherwise, heteronormative assumptions in the health and welfare sector risk contributing to inequality, contrary to what the Swedish health and welfare sector strives for. The findings indicate that there is a wide variation in the quality of healthcare.

The participants not only experienced caring encounters with professionals in the health and welfare sector; they also described uncaring encounters. Uncaring encounters included *Heteronormative assumptions*,* Intimidating behaviour*,* and Experiences of discrimination*. The participants reported experiences of heteronormative assumptions by professionals in the health and welfare sector, in line with previous research stating that people identifying as LGBTQ + encounter heteronormativity in healthcare [6–8, 12] as well as in the welfare sector [10]. Furthermore, previous research has found that the health care sector is particularly ill-equipped to deal with the unique needs of LGBTQ + individuals [31]. From a theoretical perspective, uncaring encounters are characterised by professionals being incompetent in some way, disrespectful and uninterested in the person they meet [27].

The participants stated that they were often misgendered and needed to correct professionals, and this sometimes made them avoid seeking health care. Previous research has found that sometimes, the identities that professionals ascribed to patients were misaligned with patients’ own claimed identities. These experiences contributed to reports of negative health care experiences for people identifying as LGBTQ+ [28].

The participants’ experiences of heteronormative assumptions in the health and welfare sector indicate that there is a need to improve education for professionals. The need to update documents, to be more inclusive, and allow people to describe themselves as they wish is still a matter of current debate [32]. The participants in this study wanted a respectful encounter with the use of correct pronouns. Previous research has found that health care professionals can be afraid of saying the wrong thing when meeting people who identify as LGBTQI+ [26]. The participants in this study, like those in previous research, did not want professionals to feel uncomfortable or make a big deal out of their encounters. This indicates, in line with other research [33], that professionals need time and the opportunity to reflect on norms and to practice inclusive caring encounters.

The participants also reported that some professionals showed intimidating behaviour, focusing on the wrong things. Instead of listening to participants’ reasons for seeking health care, some professionals focused on participants’ sexuality or how the participants identified themselves. These negative experiences are also reported in a previous study [32] that describes an encounter with a doctor who focused on gender identity instead of what the person had sought care for. Research also describes that individuals identifying as LGBTQ + still face inequalities and that their health needs are not met in healthcare [28, 34]. This lack of interest in the patient’s story is disrespectful and contributes to an encounter being perceived as uncaring [27]. The participants reported that some professionals asked irrelevant questions and expressed prejudice about people identifying as LGBTQ+, in line with previous research [1, 2]. Both the results of this study, as well as previous research [18], show that individuals who identify as LGBTQ + avoid seeking care due to previous experiences of poor treatment.

The participants also reported having been subject to discrimination. They experienced discrimination within healthcare when they were not allowed to donate blood or when they received the wrong type of care, as in the story where the participant was referred to a psychiatrist when they experienced gender dysphoria. Previous research reports that transexuals had to educate health care professionals about issues relating to gender identity [35]. Therefore, there is a need for explicit and consistent education for healthcare professionals and a stronger application of non-discrimination policies in clinical settings [2]. Research from the US found that experiences of discrimination were common for people identifying as LGBTQ + adults, and more than one in six avoided health care due to anticipated discrimination [36].

One participant had the experience that welfare state actors, such as the police, responded to interpersonal violence with the gendered assumption that women cannot be perpetrators. Uncaring encounters are categorised as disrespectful encounters with professionals with a lack of relevant knowledge and competence [27].

People identifying as LGBTQ + often find it difficult to navigate the health care system, and previous experiences of discrimination in health care influence their behaviour when seeking health care [37]. Professionals in the health and welfare sector are obliged to offer all individuals they meet a respectful encounter and must not subject anyone to discrimination [15, 30]. The participants’ experiences of heteronormative assumptions, intimidating behaviour and discrimination are not in line with SDG 10, which strives to reduce inequalities in health care and to promote social inclusion as well as non-discrimination.

### Strengths and limitations

The chosen method, using semi-structured interviews to collect data was suitable for exploring encounters within the health and welfare sector, and the study contributes rich descriptions of the experiences of encounters with professionals of people identifying as LBGTQ+.

Another strength is that, despite the challenges with finding participants, we managed to find quite a number with a variation in demographic background, and they represent a vast variation of the LGBTQ + spectrum. Including variety within the LGBTQ + group, can be seen as both a strength (being inclusive) but also a weakness, since the participants’ experiences can differ greatly.

The results are presented in a main category with two categories and sub-categories, with citations derived from the data, in line with the principles of content analysis. To enhance transferability, a comprehensive description of the study context, participant selection and characteristics, data collection, and analysis processes is provided. The analysis process is described in detail to meet the criteria for trustworthiness and to enable readers to follow the analysis process. Citations are used to increase the trustworthiness of the results. To ensure inter-reliability, both authors independently reviewed and coded segments of the data, followed by discussions on the formation of sub-categories under the main categories: Caring and Uncaring encounters.

## Conclusion

This study contributes to a deeper knowledge of persons who identify as LGBTQ + and their experiences of encounters with professionals in the health and welfare sector. Their experiences are included in the main category, Varying encounters in the health and welfare sector, and include both caring as well as uncaring encounters. Professionals in the health and welfare sector need to demonstrate that they support inclusion by having updated knowledge about LGBTQ + issues [38]. Therefore, professionals need to be given the prerequisites to develop guidelines for caring encounters and competence in using inclusive language.

Small things and actions can have a huge impact on the person you encounter; therefore, it is of great importance that you show an awareness of possible ways of avoiding a discriminatory approach. Every person is unique, and professionals in the health and welfare sector need to be very sensitive and open-minded with every new person they encounter. One useful strategy is to review the physical environment and make it inclusive and welcoming, something participants in the study highlighted.

Inclusion requires more than a good intention; it requires knowledge and action. In the end, caring encounters in the health and welfare sector are all about values, knowledge and respect. All people have the right to receive caring encounters in the health and welfare sector and to be treated with respect, regardless of gender expression or sexual orientation.

Organisations as well as professionals in the health and welfare sector need to update their knowledge and competence about LGBTQ + issues. More effort is needed to educate professionals in the health and welfare sector to understand individuals identifying as LGBTQ+.

More specifically, professionals within the health and welfare sector need to receive education and training about LGBTQ + individuals’ special needs and the terminology concerning them. If professionals in the health and welfare sector receive such training, their improved knowledge and competence could lead to caring encounters.

## Supplementary Information

Supplementary Material 1.

## Data Availability

The datasets used and analysed during the current study are not publicly available because they contain personally identifiable information; data are available from the corresponding author on reasonable request.

## Abbreviations

LGBTQ+: Lesbian, Gay, Bisexual, Transgender, Queer and other gender identities
SDG: Sustainable Development Goals
UN: United Nations
